# Supplementary material for: Inflammation and Prostate Cancer: Pathological Analysis from Pros-IT CNR 2
Source: Cancers (Basel). 2023 Jan 19;15(3):630. doi: 10.3390/cancers15030630 (PMC9913270; doi:10.3390/cancers15030630)
Supplement: Supplementary file 1 [file cancers-15-00630-s001.zip › cancers-2123536-supplementary.pdf]

Pros-IT2

N°	Site	Prostatic neoplasia														Chronic Inflammation													Notes
		Gleason Score									Grade Group					Anatomical location			Grade			Extent			GD		CA		
		3+3	3+4	4+3	4+4	3+5	5+3	4+5	5+4	5+5	1	2	3	4	5	1	2	3	1	2	3	1	2	3	Yes	No	Yes	No	
1																													
2																													
3																													
4																													
5																													
6																													
7																													
8																													
9																													
10																													
11																													
12																													
13																													
14																													
15																													
16																													
17																													
18																													
19																													
20																													
21																													
22																													
23																													
24																													
TOTAL																													

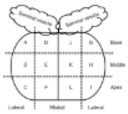

M: Left transition zone

N: Right transition zone

GD: Glandular Disruption CA: Corpora Amilacea

Figure S1. biopsy reporting scheme: the presence of PCa and its grading on the left, the presence of PI and its anatomopathological characterization on the right.
